# Cryogenic 4D-STEM analysis of an amorphous-crystalline polymer blend: Combined nanocrystalline and amorphous phase mapping

**DOI:** 10.1016/j.isci.2022.103882

**Published:** 2022-02-05

**Authors:** Jennifer Donohue, Steven E. Zeltmann, Karen C. Bustillo, Benjamin Savitzky, Mary Ann Jones, Gregory F. Meyers, Colin Ophus, Andrew M. Minor

**Affiliations:** 1 Department of Materials Science and Engineering, UC Berkeley, Berkeley, CA 94720, USA; 2 National Center for Electron Microscopy, Molecular Foundry, LBNL, Berkeley, CA 94720, USA; 3 Core R&D - Analytical Sciences, The Dow Chemical Company, Midland, MI 48667, USA

**Keywords:** Polymer chemistry, Materials science, Materials characterization

## Abstract

Understanding and visualizing the heterogeneous structure of immiscible semicrystalline polymer systems is critical for optimizing their morphology and microstructure. We demonstrate a cryogenic 4D-STEM technique using a combination of amorphous radial profile mapping and correlative crystalline growth processing methods to map both the crystalline and amorphous phase distribution in an isotactic polypropylene (iPP)/ethylene-octene copolymer (EO) multilayer film with 5-nm step size. The resulting map shows a very sharp interface between the amorphous iPP and EO with no preferential crystalline structure near or at the interface, reinforcing the expected incompatibility and immiscibility of iPP and EO, which is a short-chain branched polyethylene. This technique provides a method for direct observation of interfacial structure in an unstained semicrystalline complex multicomponent system with a single cryogenic 4D-STEM dataset.

## Highlights

• Cryogenic 4D-STEM can simultaneously map amorphous and crystalline structure
• Direct observation of beam-sensitive polymers reveals nanostructure near interfaces
• Cryogenic 4D-STEM enables mapping of chemically/structurally similar polymers in blend

## Graphical abstract

**Figure:**
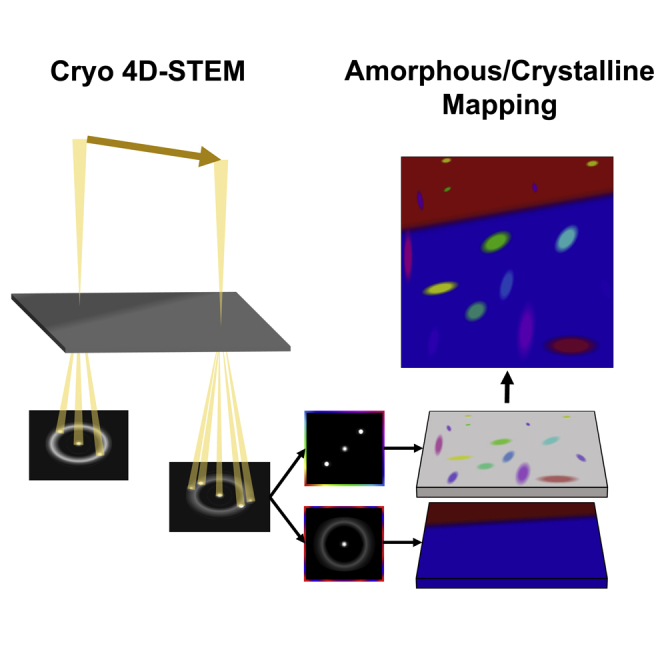

## Introduction

Polymer blends constitute a critical class of materials with application in almost every modern industry due to the impressive ability to tailor the microstructure to control properties. Most commonly these blends are optimized for a precise application by combining polymers with complementary properties to achieve a blend with properties intermediate to the component polymers (Tien and Prud’homme, 2018). However, most polymers are immiscible in blend due to a low entropy of mixing. This results in a phase-separated morphology (Salzano De Luna and Filippone, 2016), which can lead to inferior mechanical properties due to poor interfacial adhesion between the phases (Salzano De Luna and Filippone, 2016; Tien and Prud’homme, 2018). This has been widely ascribed to the same calculated thermodynamic immiscibility that produced the phase-separated morphology in the first place. The low miscibility can result in low interfacial amorphous entanglement, which leads to poor adherence and delamination between the phases (Godail and Packham, 2001).

Interfacial adhesion has motivated the study of polymers in blends, with much focus on amorphous polymer blends and adhesion mechanisms based on amorphous interactions (Salzano De Luna and Filippone, 2016; Tien and Prud’homme, 2018). However, blends very commonly consist of one or more semicrystalline polymers which generally exhibit superior strength and barrier properties compared to their amorphous counterparts (Tien and Prud’homme, 2018). In the semicrystalline blends, it has been theorized that the crystallization behavior near the interface may also significantly impact the interfacial adhesion (Chaffin et al., 2000b). To date, interfacial studies of these materials have been limited due to the considerable challenge of visualizing and characterizing these extremely localized crystalline features.

Traditionally, polymer structure has been studied by X-ray scattering, which provides average structural information at a spatial resolution that cannot examine localized heterogeneous structure at interfaces in polymers. Atomic force microscopy (AFM) provides exceptional spatial resolution and has been used considerably for phase mapping in polymers using mechanical contrast (Belikov et al., 2007; Cappella, 2011; Huda Shaik et al., 2020; Li et al., 2004), or combined spectral imaging such as nano-IR (Dazzi and Prater, 2017; Dönges et al., 2021). However, as a proximal probe technique, AFM is not readily used as a 3D structural tool to provide crystallographic information. It can be used to confirm dimensional spacings on the free surfaces of polymers that have been obtained from scattering or diffraction measurements (e.g. from SAXS or XRD) in the bulk. Even in these cases , the geometric mixing of the AFM probe shape and surface texture limits the resolution.

Transmission electron microscopy (TEM) offers the high resolution needed to observe nanoscale interfaces. However, the application of TEM to polymers is severely limited by electron beam sensitivity (Williams and Carter, 1996; Egerton, 2019), low scattering intensity from low-Z elements (Williams and Carter, 1996), and difficulty in differentiating polymers that often have close chemical and structural makeup. To circumvent this, traditionally a heavy metal stain is used to introduce artificial contrast, but this contrast can be difficult to interpret and is inherently qualitative. Additionally, staining can distort the inherent structure of the polymer, which can be crucial in understanding the local mechanics of interfaces (Libera and Egerton, 2010). In particular, heavy metal staining causes mechanical embrittlement (Brown and Butler, 1997; Montezinos et al., 1985) eliminating the possibility of *in situ* mechanical observations. Also, the staining process can introduce inorganic nanostructures (Chou et al., 2002) or produce nonlinear decoration of interfaces (Wang et al., 2007).

**Figure 1:**
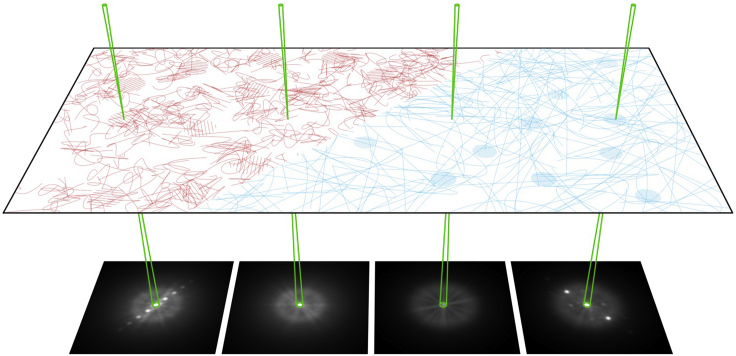
4D-STEM schematic Schematic of 4D-STEM of a semicrystalline polymer blend. A converged electron beam (green) is rastered in a 2D pattern across the sample collecting a 2D diffraction pattern at each probe position revealing the crystalline and amorphous structure with nanoscale spatial resolution. Diffraction patterns from mixed crystalline/amorphous regions illustrate the deconvolution of amorphous and crystalline scattering which allows us to map both signals independently

Recent electron microscopy instrumental and technique developments have greatly improved our ability to image unstained polymers. With the advent of high-speed detectors, four-dimensional scanning transmission microscopy (4D-STEM) has emerged as an extremely powerful technique for the collection of a wealth of information about the nanoscale structure of a material. 4D-STEM is a nanobeam diffraction technique in which a converged electron beam is rastered across the sample producing a two-dimensional diffraction pattern at each scan position as shown in Figure 1. With the recent addition of energy filtration, electron counting, and the use of cryogenic holders and a small convergence angle, 4D-STEM can now be performed with exceptional control over electron dose and provides significant improvement in signal-to-noise ratio (SNR) allowing us to collect local diffraction information from these beam-sensitive weakly scattering materials (Bustillo et al., 2021; Kanomi et al., 2021; Mu et al., 2019; Panova et al., 2016, 2019). Here, we use high-speed detectors and high-throughput processing software, py4DSTEM (Savitzky et al., 2019), to characterize both the amorphous and crystalline regions at or near interfaces with 5 nm step size.

One of the most challenging aspects of 4D-STEM is the analysis of large and complicated datasets, especially when there is scattering from multiple phases. Polymer blends are weakly scattering, and thus result in diffraction patterns with low SNR. As such, designing and implementing a high-throughput routine for extracting structural information is the most substantial and exciting challenge to implementing 4D-STEM to study beam-sensitive polymer blends. Here, we approached this challenge by first breaking the analysis into two parts which can be treated separately: (1) the amorphous structure and (2) crystalline morphology.

Isolating the weak amorphous diffraction rings and differentiating between chemically and structurally similar patterns is the first challenge, requiring careful elliptical correction, removal of crystalline contributions, and development of background removal routines. After these calibration and preprocessing steps, an isolated radial profile representing the amorphous scattering at each scan position can be obtained. From this radial profile, we map recurring amorphous structures using virtual detectors. For further insight into the atomic ordering associated with these radial profiles and phase identification, pair distribution function (PDF) analysis can be performed for further structural analysis and phase identification.

PDF analysis has a long history dating back to 1927 when the equations describing the Fourier relationship between the real-space pair density and observed X-ray scattering were first derived (Billinge, 2019; Zernike and Prins, 1927). These equations were then adapted and modified for application to electron diffraction in 1988 by Cockayne and Mckenzie (Cockayne and McKenzie, 1988) who presented a derivation of the reduced intensity function, also called the structure function. More recently, this method has been applied to analysis of 4D-STEM diffraction datasets. In this work, reduced PDF analysis was performed according to the method presented by Mu et al. (2016, 2019) with minor modifications.

The second challenge in 4D-STEM studies of semicrystalline polymer blends is the mapping of crystalline structure. Typical crystal structure analysis for diffraction patterns involves identifying diffraction peaks, calculating the associated interplanar spacing, and determining the angle of the peaks with respect to a reference axis. All this information, usually along with prior knowledge of the crystal structure, can sometimes allow for determination of crystalline orientation. However, in the case of 4D-STEM where thousands of diffraction patterns are acquired, performing this manual identification of diffraction disks is impractical. As a result, an automated data processing routine is critical for understanding the crystalline morphology associated with a 4D-STEM dataset. For high-throughput analysis of crystalline structure in 4D-STEM, often diffraction peak detection is performed using cross correlation with a probe template (Ozdol et al., 2015; Pekin et al., 2017; Savitzky et al., 2019). However, detecting diffraction disks from weakly scattering polymers with this method can be exceptionally difficult due to the low SNR. Additionally, this process can be complicated by the presence of partially illuminated peaks, which can result in poor detection accuracy in polymer diffraction patterns. To avoid these challenges, we used image correlation of neighboring diffraction patterns to reveal crystalline structures which cannot be found with disk detection.

Correlation has been historically important in electron microscopy, and image processing in general. For decades, correlation has been used for *in situ* TEM to track changes in successive images as an external stimulus is applied (Bithell et al., 1994). Generally, correlation methods have been widely utilized for tracking deformation, as well as highly sensitive orientation, and strain mapping in SEM (Littlewood and Wilkinson, 2012; Troost et al., 1993) and TEM applications (Hÿtch and Stobbs, 1994; Kiss et al., 2016; Savitzky et al., 2019; White et al., 2012). More recently, correlation has been used for matching experimental diffraction to simulated diffraction patterns for determination of thickness (Pollock et al., 2017), orientation, and composition (Ophus et al., 2017) of TEM samples using 4D-STEM. Here, we utilize correlations of neighboring diffraction patterns to reveal crystalline structures in a semicrystalline polymer blend.

The approach is to calculate the image correlation coefficient between neighboring diffraction patterns in our 4D-STEM dataset and employ a seeded region-growing technique to map continuous crystalline structures. Seeded region-growing (Adams and Bischof, 1994) is a method of image segmentation in which regions of pixels with similar values are identified. A set of pixels are first assigned as initial seeds. Neighboring pixels with sufficient similarity to the seed pixel are added to the growing region. This process is iterated until no neighboring pixels of the region are sufficiently similar. This process is repeated for all seeds. Using this method in conjunction with image correlation analysis, a technique we refer to as correlative crystalline growth (CCG), we demonstrate mapping of crystalline structure in the multiphase polymer film.

## Results and discussion

In this work, we study a coextruded multilayer film of alternating layers (of 30–600 nm in width) of iPP and EO. To study the morphology and structure near or at the interface in an iPP/EO multilayer film, the distribution of amorphous iPP/EO phases is first mapped to visualize the interface. Then, the interfacial crystalline structure is mapped using CCG.

### Amorphous phase mapping

**Figure 2:**
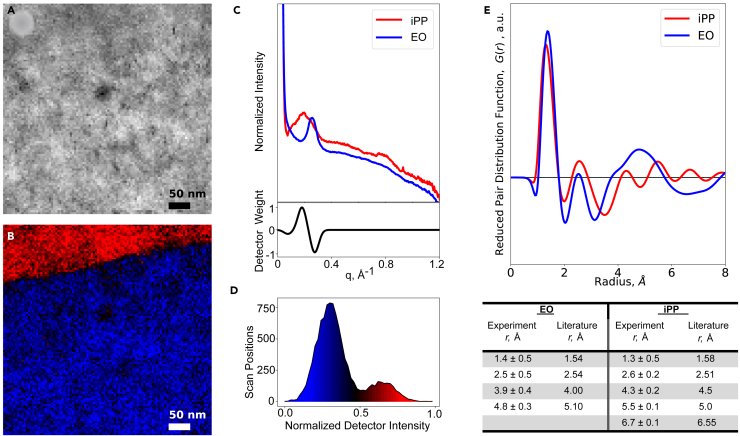
Amorphous mapping (A and B) 4D-STEM virtual bright-field image of iPP/EO multilayer film (B) amorphous phase map showing iPP(red) and EO(blue), (C) two class radial profiles representing the two amorphous phases shown in the (B) with the virtual detector utilized to make the phase map in (B), (D) histogram of the normalized intensity of the virtual detector(intensity in the phase map) versus the number of scan positions (or pixels in the phase map) overlaid with. the colormap used in (B), (E) RPDFs produced from the two class radial profiles (C), and table showing identified peaks with uncertainty originating from background fitting compared to literature (Curro et al., 1997; Narten, 1989; Wang and Yeh, 1981) reported values for polypropylene and polyethylene (see also Figure S6)

Polypropylene and polyethylene, despite being immiscible in blend, are extremely chemically and structurally similar. They both contain only weakly scattering carbon and hydrogen atoms and their structure is primarily a carbon backbone with the only difference being the addition of methyl side group in the case of polypropylene. The average densities of both iPP and EO are similar, at 0.908 and 0.902 g/cm^3^, respectively. This makes it difficult to differentiate the two using traditional TEM methods like bright field imaging which relies on mass thickness and diffraction information to produce contrast. Figure 2A is a virtual bright field image of the interface region and no contrast between the components is discernable.

From the 4D-STEM data, we directly obtained the elliptically corrected radial median profile to isolate the amorphous component of the diffraction signal (see STAR Methods). It was observed that two distinct low intensity profiles were present, one with a single apparent peak at 0.17 Å^−1^ and a second with a sharper peak located at 0.25 Å^−1^ (Figure 2C). These peaks match well to literature-expected values for amorphous polypropylene and polyethylene which demonstrate characteristic peaks at 0.175 Å^−1^ and 0.219 Å^−1^, respectively (Baker and Windle, 2001; Rabiej, 1991; Van der Burgt et al., 2002). A virtual detector (Figure 2C) was then constructed (according to method shown in Figure S2) and used to clearly distinguish between the two phases as shown in the phase map in Figure 2B. This technique exploits subtle differences in the amorphous diffraction signal to reveal a clear interface and two distinct amorphous regions. In contrast, these features are completely absent in the virtual bright-field image. A histogram of the data contained in the amorphous phase map with an overlay of the colormap used to produce the image is shown in Figure 2D. A clear bimodal distribution is observed, further validating that there are indeed two distinct classes of radial profile present in this data.

The physical meaning of these distinct profiles could then be derived using PDF analysis. The resulting RPDFs associated with each class profile are shown in Figure 2E. These were then compared to previously reported literature (Curro et al., 1997; Narten, 1989; Wang and Yeh, 1981) values for polypropylene and polyethylene as shown in the table in Figure 2e as well as calculated intramolecular RDFs from models (Figure S6) which showed good agreement within the resolution of this technique.

The sharp nature of the interface derived from the amorphous phase map likely indicates low amorphous mixing between the two polymers. This is in agreement with a large body of literature which has confirmed both the immiscible (Bertin and Robin, 2002; Chaffin et al., 2000b; Godail and Packham, 2001; Svoboda et al., 2010; Teh, 1983; Teh and Rudin, 1994) and incompatible (Chaffin et al., 2000b; Eagan et al., 2017; Fang et al., 2013; Godail and Packham, 2001; Lovinger and Williams, 1980; Strapasson et al., 2005; Teh, 1983; Teh and Rudin, 1994) nature of polypropylene and polyethylene.

### Crystalline structure mapping

**Figure 3:**
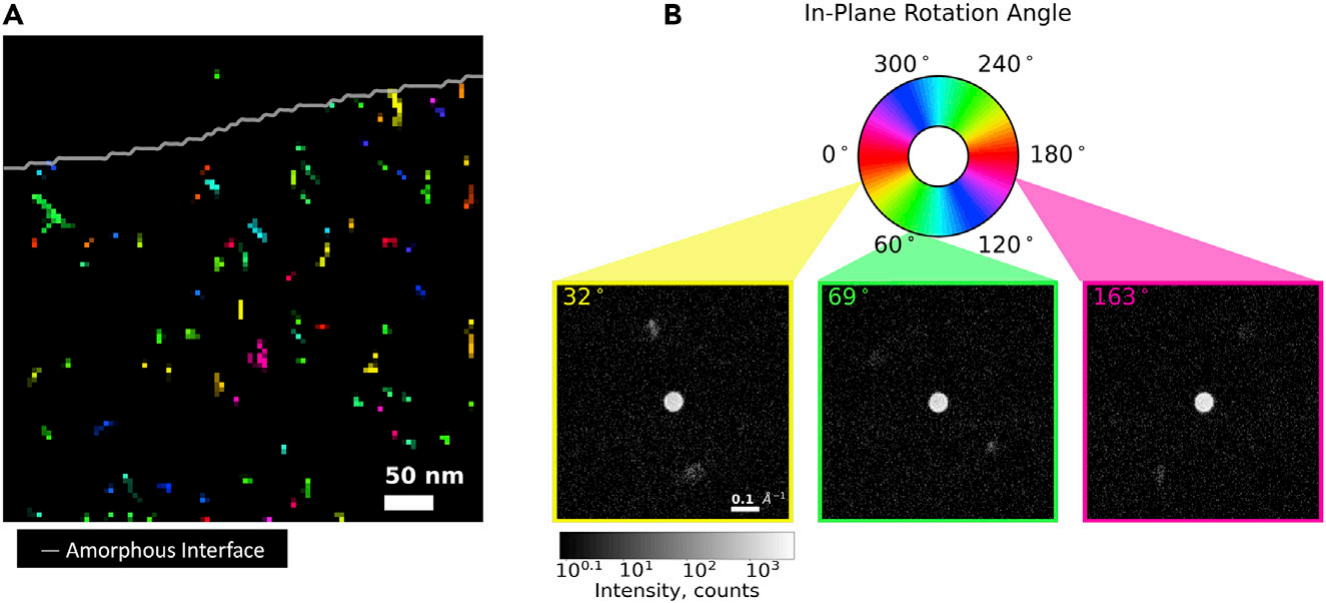
CCG mapping (A and B) (A) CCG produced map of crystal structure, (B) legend of (A) describing pattern rotation angle with three examples of high intensity seed patterns used for CCG (see also Figure S1)

The results of the CCG method (see STAR Methods) to visualize the distribution of crystalline regions are shown in Figure 3A with the amorphous interface superimposed. Crystal colors are assigned according to their in-plane rotation angle.

For determination of in-plane rotation, we identified peaks present in the seed pattern. Three example seed patterns are shown in Figure 3B plotted on log scale. Seed patterns on average contained fewer than two identifiable peaks. This is likely a result of the very low scattering cross section of these organic materials as well as the limited probability of these crystals being in a highly diffraction condition with respect to the beam. However, while the small number of peaks limited our ability to map the 3D orientations of crystallites and provided less features for the CCG method to track, it heavily simplified the determination of the in-plane, 2D rotation. From the identified peaks, we could then detect pair peaks with 180° separation and represent them by one in-plane rotation value from 0 to 180°. Once this analysis was performed, >90% of seed patterns could be represented by a single rotation angle from 0 to 180°.

In the CCG map (Figure 3), no preferential in-plane rotation near the interface was observed. Additionally, crystals did not appear more frequently at the interface. No significant variation of crystal size was observed with proximity to the interface. These results may indicate a lack of any preferential nucleation of the EO at the interface, despite the lower melting temperature of EO.

However, we note that the number of scan positions assigned as crystalline were calculated at 5% and <1% for EO and iPP, respectively. This is significantly lower than the expected 44% and 65% nominal crystallinity of the two materials. This can be attributed to several factors including low SNR, the probability of crystals being in a strongly diffracting condition, and the likelihood of beam damage causing a loss of detectable crystallinity. This is supported by the observed percent crystalline as polypropylene, being more sensitive to the electron beam than polyethylene, experienced a larger reduction in apparent crystallinity.

**Figure 4:**
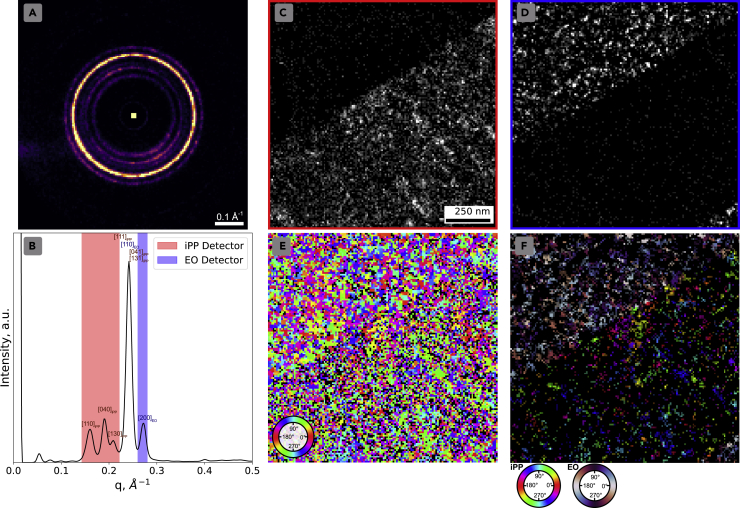
Low-dose crystalline mapping (A) the Bragg vector map from the complete dataset with associated radial integration (B) with peaks labeled corresponding to expected spacings of iPP and EO, (C and D) virtual images of iPP and EO, respectively, produced by integrating the Bragg peak intensities within the red and blue detectors in (B), (E and F) map of in-plane rotation angle of the most intense detected Bragg peak in each diffraction pattern, black indicates no peaks detected, and (F) resulting map of continuous crystalline structures using seed-region growing method. (C–F) share the same scale bar and map the same region

To determine the role of beam damage, a dataset from the same sample in a different region was obtained using significantly lower dose, 0.5 e^−^/Å^2^, and step size of 10 nm. While this provided a lower real-space resolution, this scan had a more parallel beam and a lower dose such that the SNR was significantly improved so that Bragg disks could be detected utilizing a cross-correlative template matching method. The resulting Bragg vector map is shown in Figure 4A with the accompanying radial integral in (b) with labeled planar spacings. iPP is indexed as the monoclinic *α*-form, while the EO phase is indexed as orthorhombic polyethylene. Using the virtual detectors marked in Figure 4A to integrate the identified Bragg peaks in each diffraction pattern, EO and iPP could be clearly distinguished from one another as shown in Figure 4C, and Figure 4D demonstrates effectively the expected layered structure of the coextruded sample.

With the detected Bragg peaks, we can also map the rotation angle of the highest intensity diffraction pair in each pattern and map an in-plane rotation as shown in Figure 4F. EO appears to demonstrate larger clusters of common rotation angles as opposed by the iPP phase. This work is similar to work done by Kanomi et al. (2021) where authors did rotational mapping on a neat polyethylene sample and demonstrated similar results at a 30nm step size.

However, this mapping is not a fully informed modeling of the crystalline structure as it does not consider interplanar spacing of the mapped peak and does not allow for overlapping diffraction patterns which are highly likely in a sample of this thickness and crystallinity. Therefore, CCG can provide improvement over this simplified method of classification which uses only a single rotation angle. CCG instead allows us to map continuous structures with a common interplanar spacing and rotation orientation. In this case, where peak detection does not pose such a challenge, we can perform a simplified seed-region growing technique by determining if neighboring patterns contain the same detected Bragg peaks. This method is the same in principle as CCG and similar to work done by Panova et al. (2016, 2019). The details are described in the methods section.

The resulting crystalline phase map is shown in Figure 4E. The method successfully maps continuous crystalline structure in both polymers and demonstrates significantly improved crystallinity over the scan area as opposed to the higher dose scan. In addition, to verify the sample was not significantly impacted by beam damage, a second scan with the same conditions was taken over the same area. The second scan showed little to no reduction of crystallinity as demonstrated in Figures S5 and S4.

Unfortunately, at this significantly reduced dose, the weak amorphous scattering was below the noise floor preventing detection. Therefore, amorphous phase mapping could not be performed on this dataset. Nevertheless, here, we have demonstrated the ability to map crystalline diffraction features in extremely low SNR data using CCG at a 5-nm step size and effectively shown the crystalline distribution in both iPP and EO using a significantly lower electron dose.

### Beyond 2D: statistical output

**Figure 5:**
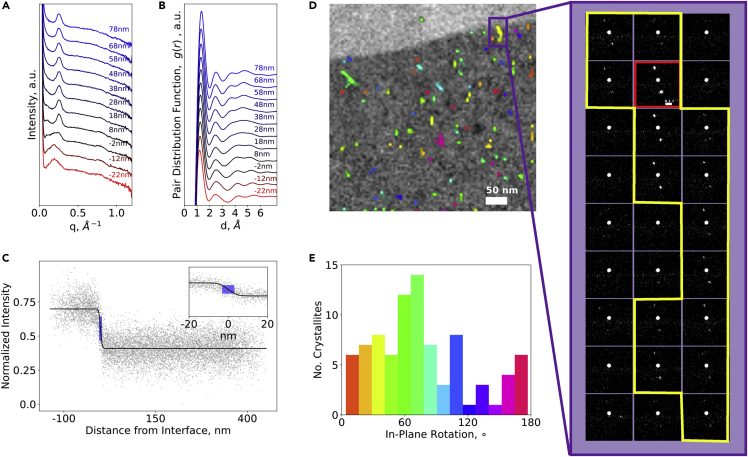
Correlated data processing (A and B) median elliptically corrected radial profile, and (B) PDF plotted as a function of distance from the amorphous interface, (C) normalized value of the amorphous phase map as a function of distance from the interface with error function fit overlaid and calculated interface indicated in blue, (D) combined CCG and amorphous phase map with zoom in showing underlying diffraction data for a single crystal in the map, (E) a histogram of the number of crystallites identified at a given in-plane rotation

Figure 5D shows the CCG map (in color) superimposed on the amorphous phase map (in grayscale) allowing direct visual comparison of crystalline and amorphous structure with the same 4D-STEM dataset. We can also directly observe the diffraction data underlying the assigned crystal regions as shown in the zoom in panel of Figure 5D.

Beyond producing a simple 2D phase map to observe nanoscale morphology, 4D-STEM along with the amorphous/CCG mapping method provides a wealth of information about local structure, which can provide meaningful statistical insights into the nanostructure of materials. Changes in the features of the PDF and radial profile can be observed over nm distances near interfaces. Figure 5B shows the variation of the PDF as a function of distance from the interface. Owing to the chemical structural similarity of these materials, there is little variation. In Figure 5A, the variation of the radial profile as a function of distance from the interface is plotted showing a distinctive change from the broad amorphous halo peaked 0.17 Å^−1^ characteristic of iPP to the narrower EO profile with characteristic amorphous peak at 0.25 Å^−1^. The stark change between the two profiles indicates a low degree of mixing, which is further supported by the sharp interface shown in the amorphous phase map. To analyze the amorphous interface, in Figure 5C, we show the normalized value of the amorphous phase map as a function of distance from the interface. From this, we fit an error function to the profile to quantify the interface width which can be linked to material properties like degree of interfacial mixing, interphase solubility, and interfacial adhesion. In this case, the interface width was measured to be 6 nm which agrees with thermodynamic calculations that predict an interface width of 4–5 nm (Chaffin et al., 2000a, 2000b; Godail and Packham, 2001). This indicates a low degree of interfacial mixing which is expected for this incompatible immiscible set of polymers.

In addition, this technique allows for the study of the statistics of the crystalline structure including crystal size, location, and rotation angle. A histogram of in-plane rotation angles (Figure 5D) showed no significant preferential in-plane orientation. We were also able to conclude there was no strong evidence of preferential nucleation or in-plane rotation angle as a function of distance from the interface (Figure S1).

This 4D-STEM technique has demonstrated an effective method of mapping both crystalline and amorphous interfacial structure in a beam-sensitive multilayer polymer film. The observed crystallinity was lower than expected, indicating that damage from the electron beam still occurred in this experiment. We demonstrated that with lower dose conditions we can map both the crystalline structure in both iPP and EO.

Future work may use an intermediate dose to the two scans. Future experiments may also alter the camera length, as it is exceptionally important as the maximum scattering angle at which data is collected determines the resolution of the PDF analysis. However, as the maximum scattering angle is increased in the diffraction patterns, crystalline diffraction peaks will occur closer to the center disk (in the region of the inelastic scattering and high-intensity atomic scattering contributions) making them significantly harder to detect. Finally, a smaller scan step size may be preferential in the future, as two key distances of interest, the crystal size and the interface width appear to be on the order of magnitude of or smaller than the scan step size used in either dataset presented here.

In summary, we have demonstrated a technique to simultaneously map both amorphous and crystalline structure in a polymer blend with 5-nm step size using cryogenic 4D-STEM in combination with median radial profile and CCG analysis. A clear interface between the phases could be detected with 6nm width, consistent with the immiscible nature and poor adhesion of iPP and polyethylene in blends. We demonstrate a crystalline mapping at two different dose conditions to assess possible effects of beam damage. No variation or preferential orientation of crystals near the interface was observed indicating no preferential nucleation of the EO on the iPP while cooling during manufacturing. This technique has opened the door to direct observation of the crystal and amorphous structure at interfaces in unstained polymer blends.

### Limitations of the study

Like most 4D-STEM work, the technique presented here requires considerable tuning of experimental parameters to acquire a dataset with viable amorphous and crystalline information while mitigating beam damage and maintaining an overall image resolution and field of view which allows observation of the feature of interest. Additionally, this work only maps in-plane rotation as opposed to a fully resolved 3D crystallographic rotation which would improve the ability of this work to draw conclusions about correlated neighboring crystallography in these samples. But, with the very recent advent of new automatic indexing algorithms (Ophus et al., 2021) for electron diffraction data, this obstacle could be overcome in future applications. Finally, as with any electron microscopy technique, as electron dose is lowered in 4D-STEM, SNR is diminished. This could theoretically limit the effectiveness of this technique in mapping structure in ultra beam-sensitive materials.

## STAR★Methods

### Key resources table

**Table:** 

REAGENT or RESOURCE	SOURCE	IDENTIFIER
**Software and algorithms**		

Py4DSTEM	https://github.com/py4dstem/py4DSTEM	https://doi.org/10.5281/zenodo.3333960

**Other**		

Isotactic polypropylene	Braskem Petrochemical Company	H375-04

### Resource availability

#### Lead contact

Further information and requests for resources and reagents should be directed to and will be fulfilled by the lead contact, Prof. Andrew Minor (aminor@berkeley.edu).

#### Materials availability

This study did not generate new unique reagents

### Method details

#### Materials

The isotactic polypropylene (iPP) used in this study was obtained from Braskem petrochemical company with a nominal crystallinity of 65% as determined by DSC and density of 0.902 g/cm^3^. The EO used in this study was ENGAGE 8540 Polyolefin Elastomer (Trademark of The DOW Chemical Company) manufactured by Dow Chemical company with a melt index of 1g/10min a density of 0.908 g/cm^3^, a glass transition temperature of −32.0 °C (Dow Chemical Company, 2011), and a nominal crystallinity of 44%. iPP and EO were coextruded to produce multilayer films with a lamellar morphology which would be easily identifiable during image processing. The resulting multilayer films had layer widths varying from 30–600nm.

This multilayer film was then sectioned using an RMC cryo-ultramicrotome at −185 °C to a nominal slice thickness of 50nm. Sections were deposited on a 200-mesh copper grid with lacey carbon support.

#### Experimental setup

Transmission electron microscopy was performed using the TEAM I microscope at the Lawrence Berkeley National Laboratory using a Gatan K3 detector and Continuum spectrometer.

A first scan was performed at −185 °C under liquid nitrogen cooling with a 300kV accelerating voltage and a semi-convergence angle of 0.5mrad which yielded a diffraction-limited probe with a full-width half-max of 2nm. The beam was rastered with a step size of 5nm over a 505 × 500nm^2^ field of view. The electron dose per sample area over the entire scan is 20 e^−^/Å^2^. However, 4D-STEM is a converged probe technique in which ∼80% of the beam fluence is contained within 1.74 × the FWHM of the probe (Bustillo et al., 2021). Using 1.74 × the FWHM of the probe as the diameter to calculate the irradiated sample area yields a dose of 60 e^−^/Å^2^ for this dataset. Gold nanoparticles were used to calibrate the reciprocal space pixel size as well as measure the elliptical distortion present in the dataset.

A second scan was taken at −185 °C under liquid nitrogen cooling with a 300kV accelerating voltage and a semi-convergence angle of 0.14mrad which yielded a diffraction-limited probe with a full-width half-max of 10nm. The beam was rastered with a step size of 10nm over a 1.4 × 1.4 μm^2^ field of view. The electron dose per sample area over the entire scan was 0.50 e^−^/Å^2^ while the dose per probe area was 0.64 e^−^/Å^2^. Gold nanoparticles were used to calibrate the reciprocal space pixel size. Elliptical calibration was performed using the diffraction rings present in the Bragg vector map shown in Figure S4.

#### Amorphous structure analysis

The amorphous regions of the polymer blend were analyzed using the elliptically corrected median radial profiles obtained from each pattern (Savitzky et al., 2021). Amorphous classification was performed using a radial virtual detector to differentiate between the two identified classes. Following this, PDF analysis was performed.

First, the radial intensity profile was obtained. To do this, each diffraction pattern in the 4D-STEM dataset was transformed from Cartesian coordinates (*q*_*x*_*, q*_*y*_*)* of the pixelated detector to polar coordinates (*q, θ*). The polar elliptical transformations were performed using built in functions of the open-source python library, py4DSTEM (Savitzky et al., 2019, 2021). This elliptical transform was used to remove the effect of stigmation introduced by the projection system of the microscope. Once the polar transformed diffraction patterns were obtained, the radial median was taken to obtain the elliptically corrected radial profile, *I(q)*, for each diffraction pattern. The median radial profile was used because in comparison to the integrated radial profile as the median is much more effective at isolating the amorphous diffraction contributions (Savitzky et al., 2019). This is based on the assumption that each diffraction pattern may include both an amorphous ring and crystalline Bragg diffraction information. To separate the two, we assume that the crystalline diffraction peaks will be sparse with high intensity relative to the amorphous ring, and thus will be best filtered out by median statistics.

A virtual detector was then utilized to distinguish between the two distinct radial profiles present in the data. To create this detector, an initial phase map was created by integrating the radial profiles between 0.139 Å^−1^ and 0.210 Å^−1^ (around the expected 0.175 Å^−1^ amorphous peak for iPP (Rabiej, 1991; Van der Burgt et al., 2002)) which showed a weak interface between the two phases. Using this initial map, a binary mask was created to approximate the location of each phase. Next, using least squares on the radial profiles with the binary image, a profile representing the variation in the radial profiles of the two regions was obtained. Following, this a virtual detector was created which highlighted the most prominent features of this profile. It is worth noting that these two peak features located at 0.17 Å^−1^ and 0.25 Å^−1^ align with the expected amorphous peak locations for iPP and EO respectively (Baker and Windle, 2001; Rabiej, 1991; Van der Burgt et al., 2002). This process is demonstrated in Figure S2.

With the obtained radial profiles, reduced PDF analysis was performed according to the method presented by Mu et al. (2016, 2019) with minor modifications and extrapolation to the PDF.

From the radial profile, the structure function, *φ(q)*, can be calculated according to,

(Equation 1) $$\varphi \left(q\right)=\frac{I\left(q\right)-Nf{\left(q\right)}^{2}}{Nf{\left(q\right)}^{2}}$$

Where *N* is the number of atoms present in the sampled volume, *f(q)* is the single atom scattering factor, *I(q)* is the intensity of the radial profile, and *q* is the diffraction angle. Here, the background $Nf{\left(q\right)}^{2}$ term is simply the addition of the single atom scattering contributions from each atom in the sampled volume. Since N cannot be determined experimentally, and the single atom scattering approximation is not valid for thick TEM samples (Egerton, 2016; Gorelik et al., 2019) (in which thickness exceeds the mean free path at a given electron wavelength), an alternative method of background fitting is used in this work. A simple power-law approximation is used to fit the underlying atomic scattering pattern and isolate the amorphous diffraction signal.

Once the structure function is obtained, the reduced pair distribution function (RPDF), *G(r),* can be obtained by taking the truncated Fourier transform of the structure function (Billinge, 2019),

(Equation 2) $$G\left(r\right)=\frac{2}{\pi }\int_{0}^{q_{max}}\varphi \left(q\right)\sin\left(\mathrm{qr}\right)dq$$

Furthermore, the pair distribution function, *g(r)*, can be obtained using a modified definition of the pair distribution function (Egami and Billinge, 2003; Egerton, 2016; Mu et al., 2016, 2019),

(Equation 3) $$g\left(r\right)=\frac{\frac{2}{\pi }\int_{\text{0}}^{q_{max}}\varphi \left(q\right)\sin\left(\mathrm{qr}\right)dq}{4\pi r}+1$$

where *r* is the real space pair separation distance. Because the number of atoms cannot be determined analytically, average density cannot be determined, we utilize the pair distribution function normalized to an average of 1 as opposed to the pair density function which would be normalized to the average material density.

#### Modeling amorphous structure

Calculation of the intramolecular radial distribution function were performed using simulation cells. The model for polyethylene was produced using the polymer modeler freely available through nanohub (Haley et al., 2018), and the model for polypropylene was produced using the monomer atomic coordinates from the nanohub polymer modeler which were then stacked in a helical structure with a rotation of 114.167° as reported in Longman et al. (1976) and Mencik (1972). The linear polyethylene models containing 40 monomer units and random back bone rotation. The iPP model contains 100 monomer units and is helical. Radial distribution function intensities were calculated according to Equation 4 reproduced from Natta et al. (1960).

(Equation 4) $${\text{A}}_{ij}=\sum_{uc}\sum_{i}\frac{N_{ij}}{r_{ij}}z_{i}z_{j}\frac{\pi }{2}$$

Where N_ij_ is the number of neighbors in the j^th^ shell about the i^th^ atom, r_ij_ is the radial distance between the i^th^ atom and the j^th^ shell, z is the atomic number of the respective atoms and uc indicates summation over the unit composition or in this case the polymer repeat unit.

#### Crystalline structure analysis

Following the amorphous analysis, crystalline structure mapping was performed using a correlative seeded region-growing technique which we will call correlative crystalline growth (CCG).

First, the polar elliptical transform is calculated. The polar transformed pattern is then Gaussian smoothed. Following this the background is subtracted from the polar transformed data to remove amorphous signal, atomic scattering contributions, and limit noise contributions. Once this is complete, the polar transform is cropped to a radial region in which diffraction is expected based on *a priori* knowledge of the materials and crystal structure (see Figure S3). These filtering parameters were manually optimized to detect the largest number of diffracted disks using a subset of manually analyzed images.

Finally, CCG is performed by first seeding crystals at locations where high intensity crystalline diffraction is identified. This identification is done by measuring the SD along the azimuthal axis of the polar transformed data. A pattern is considered significantly crystalline if the SD is more than 5 times the average across the whole scan. To avoid seeding a single crystal twice, only patterns which are local maximum in the map of azimuthal SD in a 5x5 pixel neighborhood are considered for seeding.

To perform the correlative growth the correlation, *C*_*f,g*_, of a seed to all of its nearest neighbors is calculated using the following normalized correlation coefficient equation,

(Equation 5) $$C_{f_{i,j},g_{i,j}}=\sum_{i,j}\left[\frac{f_{i,j}-\bar{f_{i,j}}}{\sqrt{\sum_{i,j}{\left(f_{i,j}-\bar{f_{i,j}}\right)}^{2}}}\cdot \frac{g_{i,j}-\bar{g_{i,j}}}{\sqrt{\sum_{i,j}{\left(g_{i,j}-{\bar{g}}_{i,j}\right)}^{2}}}\right]=\sum \hat{f_{i,j}}\hat{g_{i,j}}$$

Where, *f* and *g* are the two images being compared, and (*i,j)* are the image dimensions. If the calculated correlation is above 10%, it is considered significant, and the neighboring pixel is added to the growing crystal. The process of checking nearest neighbor correlations is then repeated for each pixel added to the crystal until no neighboring pixels to the crystal are above the threshold and crystal growth is terminated. This is then repeated for all available seeds, finally producing a map of the crystalline regions present in the sample.

In the second lower dose scan with a larger and more parallel beam, the improved SNR allowed for Bragg disk detection using a cross-correlative template matching method built into the software package py4DSTEM (Savitzky et al., 2021). Then a seeded region growing technique similar in principle to CCG was performed. First, Bragg peaks were assigned to radial and theta bins. A six pixel radial bin spacing (the width of the probe template used for matching was four pixels) and 6.3° azimuthal bin spacing were used for this purpose. In addition, all peaks at less than 0.1 Å^−1^ and greater than 0.5 Å^−1^ were removed as no expected diffraction from the monoclinic α-phase iPP or orthorhombic EO is expected beyond this region. Next, all patterns with detected Bragg disks were considered for seeding purposes. The seeds were then sorted by correlation intensity with the probe template. For each seed, the neighboring patterns were then checked to determine if they contained the same binned peaks. If neighboring patterns were found to contain one or more of the same peaks they were then added to the growing crystal. This process was repeated for each pattern adding to the growing crystal or terminated when no additional patterns could be added to the crystal. This process was then repeated for each seed. However, to prevent seeding the same crystal repeatedly, seeds were only grown if they were not already assigned to a crystal region. Growing continued until all seed patterns were exhausted resulting in a map of the continuous crystalline morphology of the sample.

## Data Availability

Cryo 4D-STEM Data Set: isotactic Polypropylene/ Ethylene-Octene Copolymer Interface https://doi.org/10.5281/zenodo.5348269 Code: Code used for this work is primarily based on open source py4DSTEM repository (https://github.com/py4dstem/py4DSTEM) and can be made accessible from the lead contact upon request. Additional information: Any additional information required to reanalyze the data reported in this paper is available from the lead contact upon request
