# Supplementary material for: Cryogenic 4D-STEM analysis of an amorphous-crystalline polymer blend: Combined nanocrystalline and amorphous phase mapping
Source: iScience. 2022 Feb 5;25(3):103882. doi: 10.1016/j.isci.2022.103882 (PMC8914558; doi:10.1016/j.isci.2022.103882)
Supplement: Document S1. Figures S1–S6 [file mmc1.pdf]

iScience, Volume *25*

Supplemental information

Cryogenic 4D-STEM analysis of an amorphous-crystalline polymer blend: Combined nanocrystalline and amorphous phase mapping

Jennifer Donohue, Steven E. Zeltmann, Karen C. Bustillo, Benjamin Savitzky, Mary Ann Jones, Gregory F. Meyers, Colin Ophus, and Andrew M. Minor

Supplemental Figures and Legends:

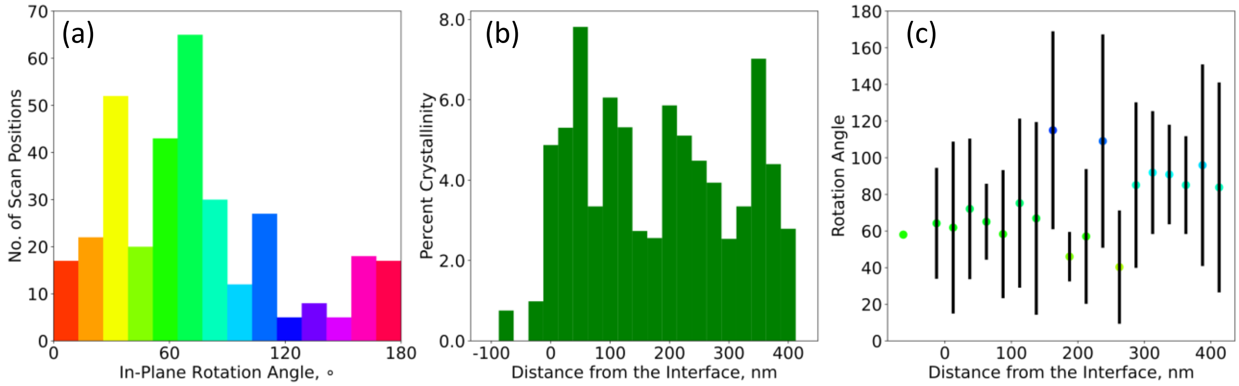

Figure S1. [Preferential Crystallization], Related to Figure 5 (a) No. of scan positions in data set versus identified rotation angle, (b) percent crystalline scan positions as a function of distance from the amorphous interface, and (c) the mean rotation angle and associated standard deviation as a function of distance from the interface.

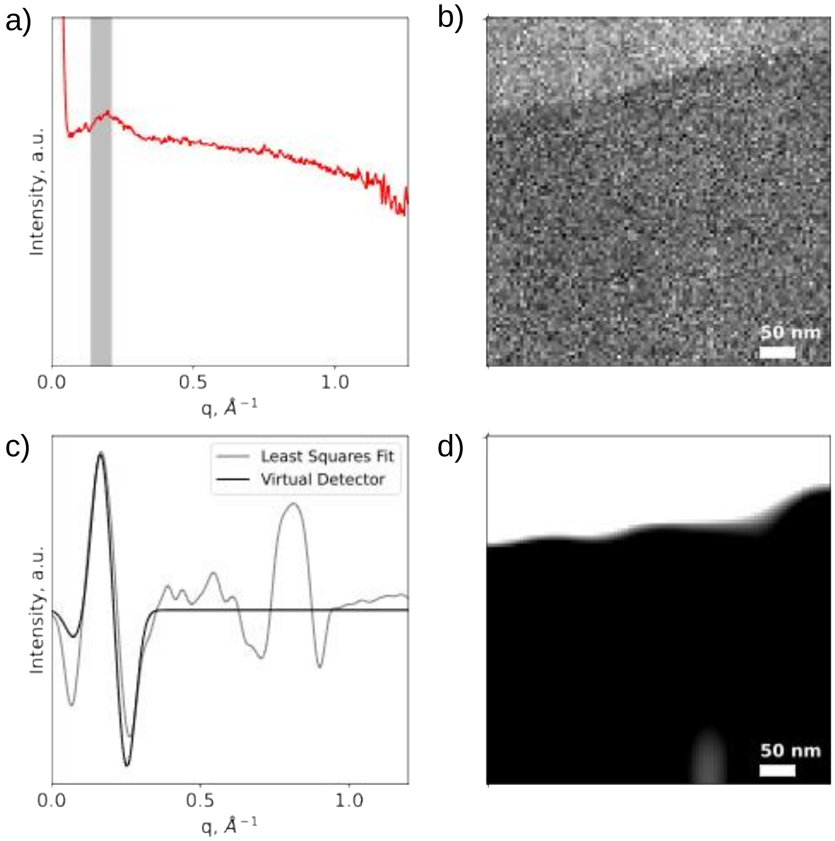

Figure S2. [Building an Amorphous Virtual Detector], Related to Figure 2 (a) example radial profile from the iPP region with integration range indicated in gray, (b) phase map produced by integration over region indicated in (a), (d) binary image produced based on (b) as preliminary phase map, (c) least squares fit for radial profiles to produce (d) with overlaid virtual detector used for phase mapping

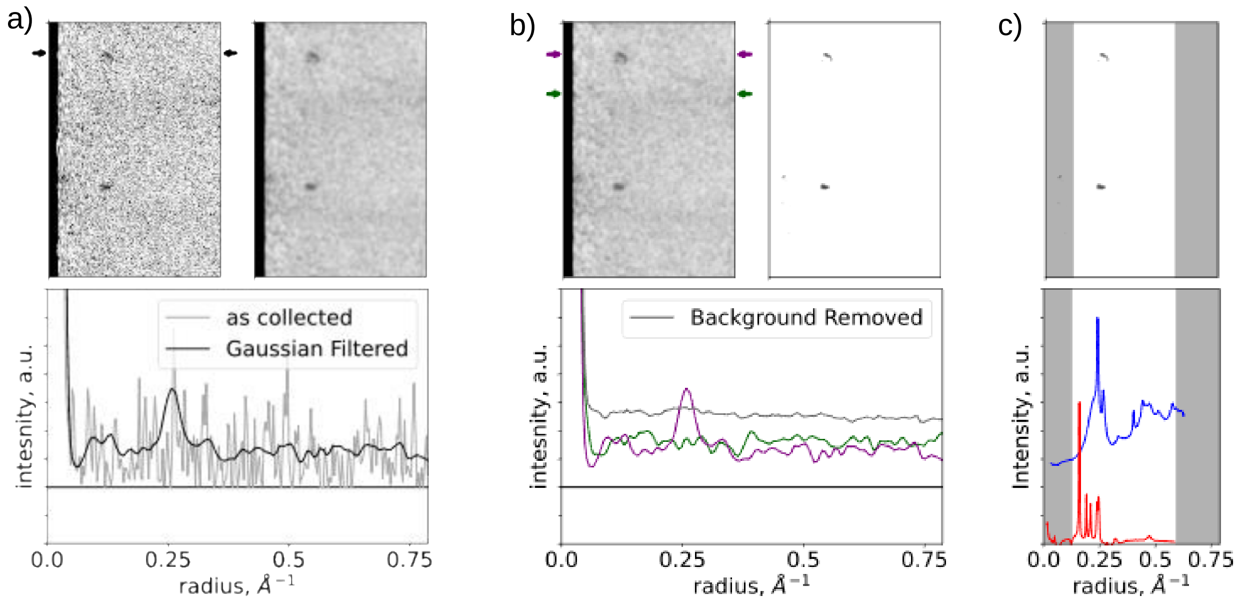

Figure S3. [Preprocessing for CCG], Related to Figure 3 (a) example polar transformed diffraction pattern before and after gaussian filtering with line slice indicated by black arrows on unfiltered diffraction plotted before and after filtering, (b) diffraction pattern before and after background removal with two line slices indicated by purple and green arrows on the initial pattern plotted below with the background used for removal, (c) processed pattern with gray region indicating cropped radial region, below is x-ray scattering data from iPP(red) and VDPE(blue) indicating all crystalline signal should be contained within the crop region

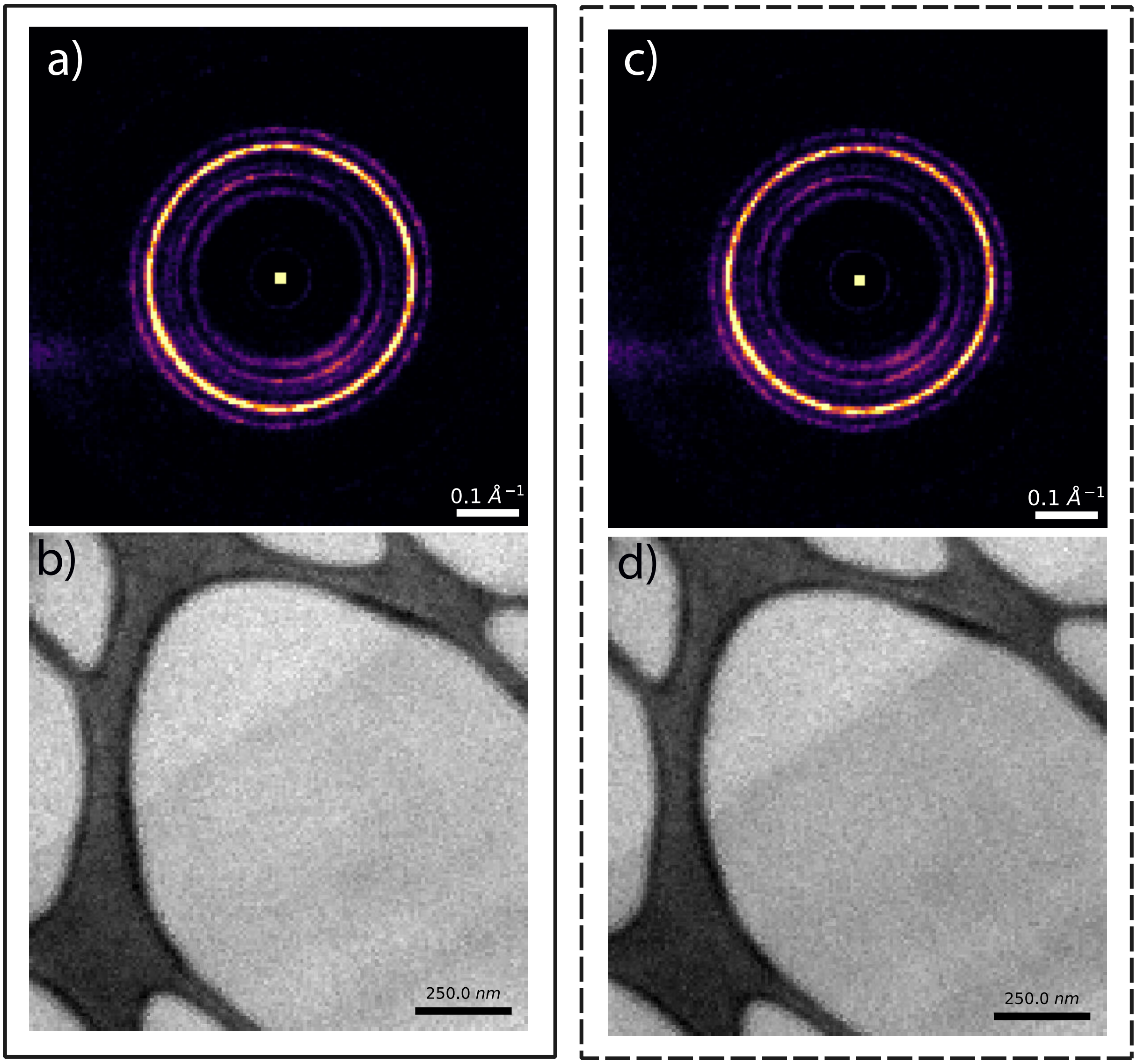

Figure S4. [Comparative Second Low Dose Scan], Related to Figure 4 (a) Bragg vector map of first scan at dose 0.5 e^{-}/Å^{2} and accompanying dark field reconstruction, (b) Bragg vector map and accompanying dark field reconstruction of same area scanned a second time producing a cumulative dose of 0.1 e^{-}/Å^{2}

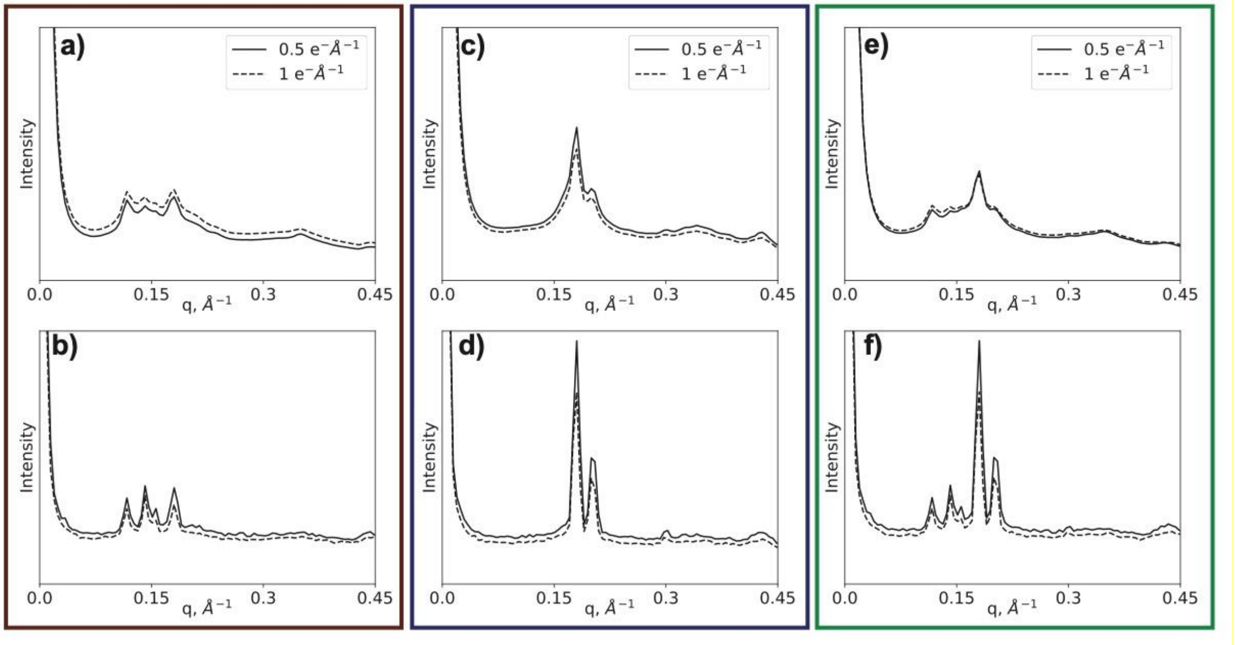

Figure S5. [Dose Comparison for Low Dose Repeated Region Scan], related to Figure 4 (a, c, e) mean radial profile of iPP region, EO region, and total field of view respectively at 0.5 e^-/Å^2 dose (solid) and 1 e^-/Å^2 dose(dotted), (b, d, f) the maximum radial profile of of iPP region, EO region, and total field of view respectively at 0.5 e^-/Å^2 dose (solid) and 1 e^-/Å^2 dose(dotted)

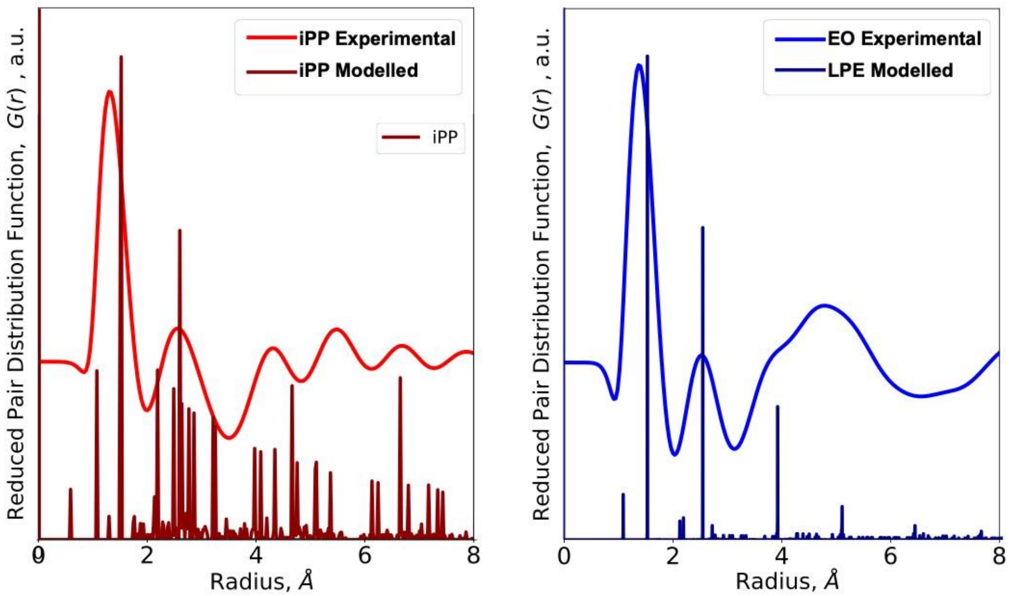

Figure S6. [Experimental vs Modelled RPDF], related to Figure 2 (a) Experimentally obtained iPP RPDF with PDF derived from intramolecular spacings of a 100 monomer helical iPP chain model, and (b) Experimentally obtained EO RPDF with PDF derived from intramolecular spacings of a 40 monomer EO chain model
